# Optical RAM and integrated optical memories: a survey

**DOI:** 10.1038/s41377-020-0325-9

**Published:** 2020-05-25

**Authors:** Theoni Alexoudi, George Theodore Kanellos, Nikos Pleros

**Affiliations:** 1 Department of Informatics, Aristotle University of Thessaloniki, Thessaloniki, Greece; 2 Department of Electrical & Electronic Engineering, University of Bristol, Bristol, UK

**Keywords:** Optical materials and structures, Optical data storage

## Abstract

The remarkable achievements in the area of integrated optical memories and optical random access memories (RAMs) together with the rapid adoption of optical interconnects in the Datacom and Computercom industries introduce a new perspective for information storage directly in the optical domain, enabling fast access times, increased bandwidth and transparent cooperation with optical interconnect lines. This article reviews state-of-the-art integrated optical memory technologies and optical RAM cell demonstrations describing the physical mechanisms of several key devices along with their performance metrics in terms of their energy, speed and footprint. Novel applications are outlined, concluding with the scaling challenges to be addressed toward allowing light to serve as both a data-carrying and data-storage medium.

## Optical memory: integration overview

Integrated optical memory technologies may in the future become an attractive option for storing data in an energy efficient and compact manner. The progress that has been made in the field has now been reviewed by three Greek researchers. Theoni Alexoudi and Nikos Pleros from Aristotle University of Thessaloniki and George Kanellos from University of Bristol describe how the energy consumption, speed and size of optical Random Access Memory (RAM) and flip-flop memory has been transformed over the past 25 years. They report how the footprint of optical memory cells has shrunk from the meter to the sub-micrometer scale, while the energy per bit has now reached 1 fJ/bit and access times have approached a few tens of picoseconds. Schemes based on semiconductor optical amplifiers, micro-ring and micro-disk lasers and photonic crystals are all discussed.

## Introduction

Over the past decades, “storing light” has appeared as a rather controversial statement, given that a photon’s inherent nature hinders its spatial confinement. The first research efforts in demonstrating optical memory functionality started as a fascinating experimental exercise, with the first optical memory being reported by means of a folded optical delay line^1^ back in 1965. Two decades later, the first optical set-reset flip-flop (SR-FF) mechanism was launched in 1985, achieving response times of <1 ns^2^, while in the next few years, research efforts mainly focused on temporarily confining light to a continuous loop inside a medium^1,3–5^. As fiber optics gradually turned into a mainstream telecom transmission platform, the research interest in optical memories experienced a significant boost in view of the possible high-speed optical signal processing applications, with a variety of schemes such as optical delay lines^6–9^, fiber-loop-based and slow-light optical buffers^10,11^ and, more recently, all-optical flip-flop (AOFF) devices being introduced for packet-level contention resolution purposes^12,13^.

Following the initial attempts to store light for packet-level processing, optical memories have made significant progress during the last decade and managed to penetrate the area of bit-level storage, significantly expanding along the performance metrics, functionality and application perspectives. This progress has been greatly facilitated by the rapid advances in photonic integration^14^ and the massive penetration of optics at interconnect segments closer to the CPU level^15,16^. At the same time, the well-known memory-related bottlenecks in the fields of computing^17–20^ and routing have served as the main motivating use-cases for transferring the speed and energy advantages of light technology to the memory domain, with the CPU-memory bandwidth bottleneck^20^ and the more recent decline of Koomey’s law^21^ comprising just two indicative examples driving research toward optical random access memories (RAMs) and optical memories for non-Von-Neumann computing paradigms, respectively.

**Fig. 1:**
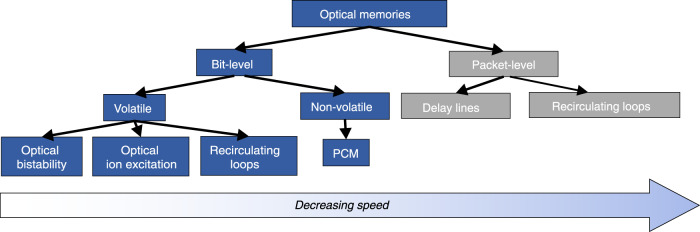
Classification of optical memory technologies

Figure 1 presents an overview of the most important categories into which current optical memories can be classified. Based on the size of the data information that is stored, i.e., a data bit or a complete data packet, optical memories can be categorized in (a) bit-level and (b) packet-level configurations, with packet-level buffering performed by more conventional and older delay line and recirculating loop technologies and as such not fitting into the scope of the current review article. Similar to electronic technology, optical bit-level memories can in turn be classified as either volatile or non-volatile structures, depending on whether the stored data are lost or maintained, respectively, when the power supply is switched off. Optical volatile memories can typically offer faster access times and higher speed operation compared to their non-volatile counterparts and form the core memory mechanism in the optical versions of the well-known and highly useful RAM cell architectures, again discriminated into two main categories: (a) the optical dynamic (DRAM) and (b) the optical static (SRAM) RAM, with their main difference lying in their requirement for refreshing (DRAM) or not (SRAM) the stored bit value. Optical SRAM layouts have thus far been implemented mainly by means of bistable optical devices^22–43^, whereas the optical DRAM cells that have been reported rely on either low-speed optical physical mechanisms such as ion excitation^44–47^ or recirculating loop arrangements^48,49^. Optical non-volatile memories are a more recent addition to light-enabled memory technology, mainly taking advantage of the rapid progress experienced in the field of phase-change material (PCM) structures^50–55^, which have been shown to allow for permanent light storage in a continuously growing field of diverse applications.

In this article, we review the substantial progress witnessed in the field of integrated optical memory technologies, mainly focusing on bit-level volatile and non-volatile optical structures and on roadmaps for transforming these elementary optical memory modules into practical optical RAM cell layouts. The paper is organized as follows: First, the basic approaches and principles applied to achieve light-based storage in general are presented, and the main technical system requirements in terms of memory are discussed. Then, the state-of-the-art optical memory technologies and concepts are reviewed, and their benefits in terms of energy, bandwidth and footprint are summarized. Following this, advanced memory functionality i.e., true optical RAM operation, is explained, and recent advancements are reported. Finally, an analysis is presented for the next steps that optical memory technologies must undertake to release a viable and practical alternative memory roadmap.

### How to store information with light

**Fig. 2:**
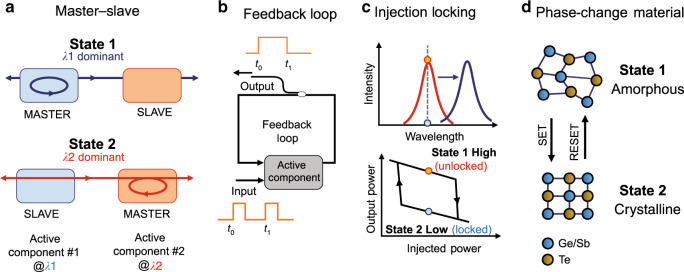
Optical memory bistability based on different approaches. **a** master-slave scheme, **b** feedback loop scheme, **c** injection-locking technique and **d** phase-change material (PCM) properties in the case of GST compounds

Although light has inherent disadvantages when considering buffering functionalities, as the neutral charge of photons makes it impossible to mimic the respective capacitor-based electronic memory layouts, the research community has devised several methods to enable light-based storage. The most common approaches to achieving this rely on either the bistability of engineered optical resonances (artificial cavities), such as in refs. ^22–36^, or the inherent bistable characteristics of devices stemming from their material properties^37–43^. Two main conditions should be applied to achieve optical bistability and consequently memory operation: the system should (a) provide at least two discrete, stable states that represent the logical one and logical zero and (b) allow switching between the two states under certain conditions. Figure 2 summarizes the four most popular categories of bistable memory devices, which rely on (a) the master-slave configuration, (b) the feedback loop scheme, (c) the injection-locking technique and (d) phase-change materials. In the master-slave configuration (Fig. 2a), two active components that are usually either switches^22,24,30^ or lasers^30^ are placed in a coupled arrangement, forming an artificial cavity. In this case, the discrete memory states (i.e., logical value “1” or logical value “0”) are represented by two different states of a certain light beam characteristic, such as the polarization or wavelength of the light beams emitted by the respective active components.

Figure 2a depicts the case where two different wavelengths are used to denote the different binary states. Each time, only one of the two available wavelengths can be dominant in the cavity, whereas the other remains suppressed. Assuming that wavelength λ_1_ corresponds to the logical value of “1” and wavelength λ_2_ represents the logical value “0” emitted by active component #1, State 1 refers to the cavity situation where light at λ_1_ dominates the cavity and suppresses wavelength λ_2_ emitted by active component #2. As long as State 1 is dominant, active component #1 serves as the “master”, whereas active component #2 is the “slave”, with the memory output signal obtained at wavelength λ_1_. Conversely, in State 2, wavelength λ_2_ suppresses wavelength λ_1_, and the memory output emits a signal at wavelength λ_2_. Changing between the two states is accomplished by injecting external light at the appropriate amount of power and wavelength into the “master” component, suppressing its operation and allowing sufficient time for the “slave” device to recover to its equilibrium state. In this case, the wavelength emitted by the “slave” device can then reach the “master” device, acting as a holding signal that retains the suppression of the former “master” wavelength even if the external light injection stops. This type of scheme is usually employed for set-reset flip-flops (SR-FFs), which have also been employed in optical SRAM cells^22,23^. To date, theoretical studies on coupled schemes^56^ have revealed that the switching time between two states is inversely proportional to the length of the cavity formed between the two active components, suggesting that an integrated solution has to be adopted to enable switching times in the picosecond regime.

Optical memories based on feedback loops, shown in Fig. 2b, require a single active component along with an external cavity usually implemented by loop configurations that feed the output signal back to the active element either through a fiber^26,27,48,49^ or by using an integrated bent waveguide^36^. The cavity acts as the memory element, enabling bit storage, and a tap of the cavity allows for monitoring the logical state of the feedback loop, i.e., the memory content. The active element employed so far is a 1 × 2 optical switch^26,27,31^ that either feeds the loop with the switched signal supporting its recirculation or blocks the recirculation by switching the signal out of the loop. This type of optical memory has been demonstrated in SR-FF schemes^33^ using independent and discrete set and reset externally injected signals but has also been employed to build toggle flip-flops (T-FFs)^26,27,31^ by applying a single external pulsed signal, as depicted in Fig. 2b. The demonstrated T-FFs^26,27,31,32^ follow an electronics-borrowed approach where two options are available: (a) either maintain the current state's value for another cycle in the case of a logical zero or (b) toggle the value (negate it) at the next clock edge in the case of a logical one at each input. In that case, the loop retains its state when the incoming signal is blocked; otherwise, the memory content is changed, yielding a T-FF functionality that is highly useful for shift registers and counters^32,57^.

On the other hand, the injection-locking technique widely used in lasers can provide optical memory bistability^37,42,43^ by forcing specific light characteristics of the lasing device to lock to the respective characteristics of an externally injected optical beam. The light characteristics that can be applied through the locking mechanism are usually the (a) wavelength^37–39,42,43^, (b) polarization state^40,41^, and (c) propagation direction^30,33,35^. In this case, memory bistability is observed as the interchange of the laser emission states between a free-running mode (unlocked/high state) and an injection-controlled mode (injection-locked/low state). The principle of operation in the case of wavelength bistability is graphically represented in Fig. 2c. Initially, the laser emits at its free-running state signal, shown in red in Fig. 2c. In the presence of a wavelength-detuned input injection signal, called the control signal, the laser starts emitting at the injection wavelength (blue in Fig. 2c) and not at its free-running mode wavelength when the control power increases above a specific threshold. As the optical power of the control signal decreases to a certain value at this state, the device enters a hysteresis loop retaining this emission state even when the control signal optical power is decreased to a certain cut-off level. Figure 2c illustrates an indicative hysteresis loop formed by a laser device assuming a given wavelength detuning. As is evident, when the optical power of the injected signal falls below this cut-off level, the laser emission returns to its free-running “unlocked” state. Consequently, the laser emission output has two states, i.e., locked (low) and unlocked (high), which depend on the ascending or descending direction of the injection signal power, and the memory operation can be achieved when operating within the bistable range of the laser device. Similarly, memory bistability can be achieved by means of polarization^40,41^ by interchanging the polarization state (orthogonal or vertical polarization) of the injected optical signal, while in the case of the propagation direction, the light in the device can be forced to circulate either to the clockwise (CW) or to the anticlockwise (ACW) propagation mode^30,33,35^ by setting the injected external signal in the appropriate direction.

Another approach to enabling optical memory bistability relies on the exploitation of the physical properties of the optical phase-change materials (O-PCMs)^52–55^. O-PCMs have emerged as a unique class of materials that can exhibit large changes in their optical properties (index change Δ*n* > 1, Δκ ~ order of magnitude) in response to an external stimulus (i.e., temperature, applied voltage or ultra-fast optical excitation). Most established O-PCMs for optical memories are chalcogen-based alloys such as Ge_2_Sb_2_Te_5_ (commonly known as GST), in which the material undergoes transitions between its amorphous and crystalline states. An example of the principle of operation of a PCM-based optical memory is shown in Fig. 2d. In this recently introduced all-optical PCM memory^52^, a small patch of GST loaded on top of a silicon-nitride waveguide is used, and memory bistability is triggered by injecting optical pulses that can lead the thin film to adopt either an ordered crystalline or disordered amorphous state. In Fig. 2d, different colors represent different atoms, such as Ge, Sb, and Te, in the GeSbTe compound^52^. The phase of the GST element affects the optical properties of the underlying waveguide such that the specific phase and subsequently the memory content can be concluded by monitoring the intensity of the propagating light at the output. In the crystalline state, the GST is more absorptive, inducing strong attenuation to the propagating light, which results in low intensity at the output that corresponds to the logical “0”. On the other hand, in the amorphous state, the absorption is reduced, allowing for high-intensity pulses at the output and yielding a logical value of “1” at the memory output. Switching between the two phase states occurs when high-intensity optical pulses are injected and, based on their total energy, can initiate either amorphization (write) or crystallization (erase). It is important to note that this type of memory element can also be configured to support multiple intermediate absorption levels between its two extreme states, allowing for multi-level operation and multi-bit storage properties.

### State-of-the-art optical memory technologies

**Fig. 3:**
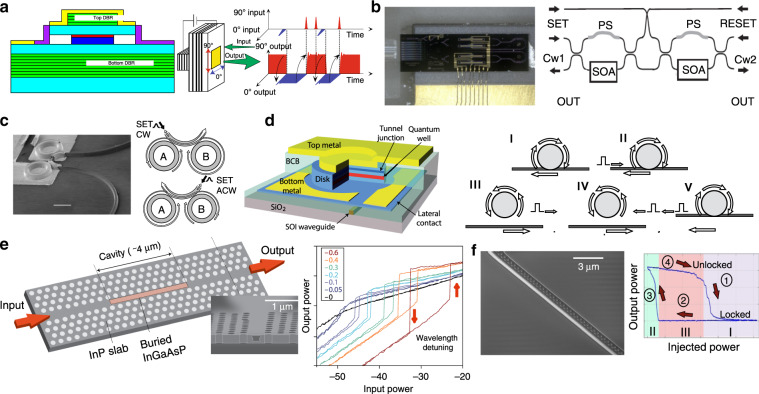
Optical memory devices and their respective principle of operation. **a** a VCSEL-based optical memory, **b** an SOA-MZI coupled optical memory, **c** an InP micro-ring laser memory, **d** an InP microdisk memory, **e** an InGaAsP photonic crystal nanocavity memory and **f** an InP-on-SOI hybrid photonic crystal nanocavity laser memory. Figure reproduced with permission from **a** ref. ^40^, © 2010 OSA; **b** ref. ^25^, © Photonics Journal IEEE; **c** ref. ^30^, © 2004 Nature Publishing Group; **d** ref. ^35^, © 2010 Nature Publishing Group; **e** ref. ^38^, © 2012 Nature Publishing Group

**Fig. 4:**
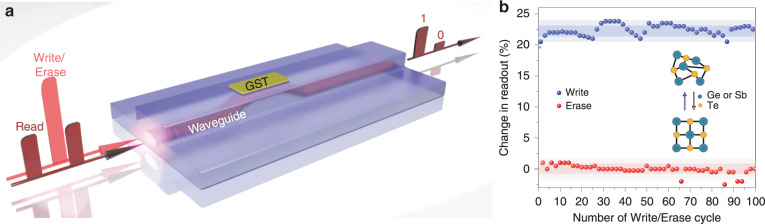
Optical phase-change memory device and its respective principle of operation. **a** Information is stored in the phase state of the GST section on top of the nanophotonic waveguide. **b** Demonstration of binary memory operation between the crystalline (lower, level 0) and amorphous (upper, level 1) states of a 5 μm GST device for multiple repetitions of the same switching cycle. Figure reproduced with permission from ref. ^52^, © 2015 Nature Publishing Group

In this section, we review the current state-of-the-art optical memory technologies. Figure 3 summarizes the most popular optical volatile memory technologies that have been successfully pursued toward delivering light-based storage, relying mainly on (a) VCSELs, (b) semiconductor optical amplifiers (SOAs), (c) InP coupled ring lasers, (d) an InP microdisk laser, (e) InP buried heterostructure (BH)-PhC nanocavity switches, and (f) hybrid InP-on-SOI PhC lasers. Figure 4 presents the non-volatile PCM optical memory technology platform and its main principle of operation.

#### Vertical cavity surface emitting lasers (VCSELs)

The first optical memory demonstration based on a bistable vertical cavity surface emitting laser (VCSEL) was reported in 1991 58. Since then, significant research efforts have been invested toward achieving VCSEL-based memories^40,41,58–61^. The VCSEL structure for polarization bistability at the 1.55 μm wavelength region and its respective principle of operation are shown in Fig. 3a. It exploits the polarization bistability, which is controlled through the injection of an external signal with an appropriate power and polarization direction (orthogonal or vertical), such that the polarization state of the VCSEL output signal follows the polarization state of the injected optical pulse. In this way, the memory content (i.e., logical state “0” or “1”) of the VCSEL optical memory is identified based on the polarization state of the output signal. Polarization bistable 980 nm VCSEL-based memories have been demonstrated with 20-Gb/s RZ and 40-Gb/s NRZ optical pulses 40 at slower repetition periods, enabling multi-bit memory implementations^61^. The main advantages of polarization-bistable VCSELs include (a) their potential for high-speed memory operation^62^, being able to handle up to 40 Gb/s optical pulses, (b) their attractive properties for logic gate functionalities^63^, (c) their low-energy consumption requirements (~105 fJ for 40 GHz operation^40,41^) compared to other types of bistable laser diodes, stemming from their lower bias current requirements^63^, and (d) their established and mature laser technology platform, which can form the basis of a reliable optical memory solution. Among their main limitations are certainly the relatively increased footprint requirements, as only the active square mesa corresponds to ~36 μm^2^^62^, and the need for a carefully controlled polarization state and alignment, especially when targeting multi-bit integrated modules.

#### Semiconductor optical amplifier (SOA)-based schemes

In the early 2000s, several AOFF demonstrations were introduced relying on semiconductor optical amplifier (SOA)-based lasers or switches performing in master-slave configurations and exploiting well-known non-linear physical phenomena such as gain saturation^64,65^ and polarization-dependent gain saturation in SOA-based switches^66,67^. These demonstrations were implemented with discrete fiber-pigtailed components, with the first integrated AOFF appearing in 2006^68^. This AOFF utilized hybrid silica-on-silicon integration technology and a coupled SOA-MZI-based architecture 68 and mainly targeted all-optical packet switching applications to facilitate routing and forwarding directly in the optical domain^12,13^. Bit-level optical memory implementations were demonstrated shortly after utilizing cross-phase modulation (XPM) phenomena in SOA-MZIs^22^, cross-gain modulation (XGM) in coupled SOAs^23,24^ or SOA-based coupled ring lasers^32,69^. The transfer of this AOFF scheme into its InP-based monolithically integrated version, which was then also employed in true optical RAM cell setups, was only recently demonstrated^25,70^, reporting 10 Gb/s operation and a drastic footprint reduction of 97.8%% compared to its hybrid-integrated predecessor^68^. A photo of the monolithic integrated device and its principle of operation are shown in Fig. 3b, illustrating that it follows a master-slave configuration, with the two coupled SOA-MZIs being powered by two external continuous-wave (CW) input signals CW1 and CW2 and the logical value of the memory cell being determined by the wavelength of the dominant CW signal. Other AOFF schemes based on SOA-based DFBs^71^, SOAs in combination with DFB laser diodes^72^, loop mirror setups^73^, and feedback loops^34,36^ have also been presented. Among the main benefits of SOA-based technologies in optical memory implementations^22–27,34,36,49,64–73^ are (a) their enhanced maturity level and flexibility characteristics that, in many cases^22–24^, allowed for the proof-of-concept demonstration of novel memory concepts prior to proceeding to their more compact and integrated versions^25,36^ and (b) their high-speed potential, having already resulted in 10 Gb/s memory line rates^70^ and being theoretically predicted to allow up to 40 Gb/s operating speeds even in optical SRAM cell arrangements^56^. However, their energy and footprint drawbacks probably critically affect its practical perspectives: SOA-based AOFFs require very large amounts of energy for both SOA biasing (~120 pJ^25^ and ~180 pJ^70^) and for optically switching between set and reset states (~3 pJ^25^, ~0.5 pJ^70^), with the current footprint requirements hardly going below a few mm^2^.

#### (Micro) ring lasers

A fast, low-power AOFF-integrated memory based on coupled micro-ring lasers exploiting the injection-locking technique was reported 30 in 2004, with its principle of operation shown in Fig. 3c. By connecting two ring lasers together via a waveguide, as depicted in Fig. 3c, two inherent lasing modes can be exploited to create a system where the master micro-laser injection locks the slave laser under certain conditions and defines the direction of the propagating light, dictating in this way two possible stable states: (a) laser light traveling in the clockwise (CW) direction and (b) laser light in the anticlockwise (ACW) direction. To switch states, light close to the lasing characteristics in terms of the wavelength and polarization needs to be injected into the waveguide connecting the lasers to set both lasers to lase simultaneously in either the CW or ACW direction. Alternative AOFFs and optical memory demonstrations relying on semiconductor ring lasers have also been suggested^33,74,75^ following the rationale that the cavity should support two counter-propagating directional modes. The first demonstration was a novel single semiconductor micro-ring laser employing a retro-reflector cavity to enable 2-bit optical storage while achieving fast ON/OFF switching times^74^. Another semiconductor ring laser was also proposed by CNIT in 2013, who reported high-speed operation at 10 Gb/s and an improvement in the switch-ON times up to 10 ps^33^. The proposed micro-ring lasers^30^ can provide electrically pumped optical memory implementations, also requiring, however, an additional DC current bias to tune the resonant frequencies of two lasers close to each other^30^. Although integrated ring laser schemes can offer some attractive advantages such as (a) multi-Gb/s operational speeds (10 Gb/s 33) and fast switching times of 20 ps^28,30^ and (b) high-output-signal extinction ratio values that can reach almost 40 dB^74^, their main drawbacks remain (a) the total energy consumption accounting for several pJ (~1.2 pJ^30^ and 54 pJ^33^) and being mainly dominated by the bias current (30 mA^30^, ~200 mA^33^) and (b) their large footprint, occupying more than 1000 μm^2^ ^74^ and reaching, in some cases^33^, even several mm^2^.

#### Microdisk laser

An ultrasmall, low-power, electrically pumped AOFF memory on a silicon chip was introduced by IMEC in 2010^35^. The AOFF relied on a single microdisk laser with a diameter of 7.5 μm coupled to a silicon-on-insulator (SOI) wire waveguide. Figure 3d shows a schematic of the microdisk laser and its principle of operation, which again exploits the propagating light direction to designate an AOFF state, relying on the interchange between the clockwise (CW) and anticlockwise (ACW) propagation directions of the whispering gallery modes (WGMs) supported by the microdisk. Assuming that the microdisk laser works initially in the CW dominant state (Fig. 3d–I), the ACW mode is suppressed, and the optical power measured at the left side of the SOI bus waveguide is high. When an optical reset pulse is injected (Fig. 3d–II), it will invoke the ACW mode, which will be retained even after the reset pulse has passed through the microdisk laser, as shown in Fig. 3d–III. In this case, the power monitored at the left side of the SOI waveguide becomes low. Switching back to the CW dominant state can be achieved by injecting an optical set pulse from the right side of the SOI waveguide, as shown in Fig. 3d-IV,V^35^. Microdisk-laser-based memories comprise a highly compact integrated memory scheme that has low switching power requirements (1.8 fJ^35^) and fast switching times (~60 ps^35^) but requires additional power for thermal tuning (~0.8 mW/bit^35^) that increases the total energy consumption.

#### InP buried heterostructure (BH) photonic crystal (PhC) laser/nanocavity

Significant research efforts have been invested in recent years in investigating (a) InP BH-PhC lasers^37^ targeting all-optical signal processing and next-generation optical packet switching systems and (b) nanocavities^38,39^ toward achieving successful optical memory operation for various types of optical processing, including network routing. In 2011, the first optically pumped PhC laser-based AOFF was introduced relying on a wavelength injection-locking technique in an InGaAsP/InP BH-PhC laser that exhibited fast switching times of 60 ps and switching powers in the range of ~20–70 μW^37^. A significant step in the advancement of optical memory was performed in 2012, when a BH-PhC nanocavity again integrated in InGaAsP platform material was used to demonstrate optical memory bistability with a record-low static energy consumption on the order of 30 nW^38^. Figure 3e shows a cross-sectional electron micrograph image of a fabricated sample, the respective hysteresis response when the laser wavelength was detuned by an offset *d* from its resonance, and the output power (*P*_out_) versus the input power (*P*_in_) for different wavelengths. The proposed BH-PhC nanocavity memory was tested with short pulses that can, in principle, lead to attractive memory speeds of 40 Gb/s^38^; however, the switch-OFF time reported was on the order of 7 ns owing to the slow carrier relaxation time in the cavity. This technology was also the first to demonstrate high-integration-density memory setups exploiting wavelength-division-multiplexing^39^ and yielding a 128-bit storage capacity^39^. Recently, an InP photonic crystal nanocavity with an embedded InGaAsP active region demonstrated an all-optical memory with only 2.3 nW operating power requirements^76,77^ and unlimited storage time. Among the most important advantages of the InP BH-PhC nanocavity-based memory technology are certainly (a) the ultra-low-energy consumption and (b) the proven capability to produce multi-bit photonic memory chips and high integration, with the main drawback thus far being the rather long switch-OFF time, which has most likely restricted their application to high-speed data traffic.

#### Hybrid InP-on-SOI photonic crystal (PhC) laser

Photonic crystals (PhCs) represent a disruptive solution toward low-power nanophotonic circuitry, with the heterogeneous integration of PhC lasers having also been successfully employed for optical memory operation^42,43,78^. The first InP-on-SOI PhC laser-based memory setup was demonstrated for the first time in 2013 using an optical pumping scheme and reporting on the >2 s storage capability^78,79^. More recently, this laser structure was shown to perform successfully even with true pseudo-random bit sequence (PRBS) data patterns in both fundamental logic functionalities, i.e., gating^77^ and latching^42^. Figure 3f shows the PhC nanocavity laser device and its principle of operation when relying on wavelength bistability through injection locking, depicting an indicative hysteresis loop formed for a given wavelength detuning. The device requires a constant optical bias signal and operates as a set-reset AOFF, taking advantage of the three discrete areas of injection signal optical power levels, as shown in Fig. 3f: Area I, where the injection power levels allow for the set operation, as the laser output is changed from a free-running (unlocked) to an injection-controlled (locked) state; Area II, where the injection power levels are below a certain threshold, enabling the reset operation, i.e., the laser output returns to its free-running (unlocked) state, and Area III, where the injection power levels cover the bistable range and enable the storing operation, because the laser emission retains its previous state. Hybrid InP-on-SOI PhCs combine some important advantages for memory applications as they can satisfy at the same time three critical requirements: (a) low footprint (6.4 μm^2^), (b) low-energy consumption (13 fJ) and (c) high-speed bit-level operation, which have all been already verified experimentally at up to 10 Gb/s with the true data traffic^43^. Considering that this memory technology can, in principle, be migrated to an electrically pumped scheme similar to the respective electrically pumped PhC laser nanocavities demonstrated more recently^80^. This platform seems to hold all the necessary credentials toward promising optical memories for real application needs.

#### Other optical bistable memory technologies

Several optical memory demonstrations employing bistable laser diodes^81–84^ and injection locking in Fabry–Perot (FP)^85,86^, DFB^87^ or DBR lasers^88^ have also been presented. Memory schemes with V cavity^89^ and modulated-grating Y branch^90^ lasers as well as symmetric Mach–Zehnder switches with 2D photonic crystals were also exhibited^91^. Nevertheless, the majority of these designs are relatively complex or require difficult active-passive integration techniques or high currents.

#### Ion excitation and recirculation loops

In addition to the volatile optical memory schemes that have been analyzed thus far and rely on some type of bistability-induced latching mechanism, there have also been alternative volatile optical memory layouts demonstrating the exploitation of a storage mechanism closer to the principle of electronic capacitor-based memories. Erbium-doped^47,92^ or erbium–ytterbium-doped^45^ fiber absorption and fluorescence properties take advantage of the optically induced Er ion excitation to store information in the form of an excited state, which, however, decays after a few milliseconds, resembling the behavior of the electronic capacitor discharge. Identifying this correlation, these schemes have been successfully proposed for mimicking electrical dynamic random access memory (DRAM) circuitry and demonstrating respective optical DRAM cell layouts^44–47^. Similarly, alternative volatile optical memories for optical DRAM setups were demonstrated using SOA-based fiber loops^48,49,93^, where again a memory refresh operation had to take place by re-writing the bit that circulates in the loop and inevitably had its quality degraded after a certain amount of recirculation. Although these layouts comprise scientifically interesting attempts to transfer electronic DRAM functions directly in the optical domain, their fiber-based implementation thus far comprises a significant limiting factor in their practical application perspectives, revealing a rather limited maturity and increased power consumption requirements.

#### Optical phase-change material (O-PCM) memories

Optical phase-change materials (PCMs) have emerged in recent years as a unique class of material platforms with great promise for non-volatile integrated photonic memory applications^50–55^. PCMs can switch between the amorphous and crystalline phases by applying only short optical pulses with very low energy, being able to retain their new state for very long times. At the same time, their amorphous or crystalline state is associated with different light absorption levels, allowing their translation into optical memory functions by simply encoding the stored material phase into the power level of propagating light. The transition between the two phases can be performed on a picosecond to sub-nanosecond timescale for amorphization and on a sub-nanosecond to nanosecond timescale for crystallization. In 2015^50^, an all-photonic, non-volatile memory based on Ge_2_Sb_2_Te_5_ (GST) phase-change material was proposed for the first time, performing at 1 GHz optical clock frequencies and allowing for all-optical multi-level and multi-bit memory capabilities. As illustrated in more detail in Fig. 4a, information is stored in the phase state of the GST section that is placed on top of the nanophotonic waveguide. Both reading and writing of the memory can be performed with ultrashort optical pulses, utilizing the interaction between the evanescent field of the guided light and the GST material. During the read operation, a weak optical probe pulse is employed to obtain the material phase encoded onto the power level of the probe pulse, while the write and erase operations require the use of intense optical pulses that are responsible for enforcing a phase transition within the GST. As shown in Fig. 4b, the crystalline state of GST (level 0) results in higher absorption levels and, as such, increased attenuation compared to its amorphous state (level 1). As such, the data are stored in different material phases, finally translating into a different attenuation factor, allowing its content to be finally read at the amount of probe light transmitted through the waveguide. This principle has been utilized in ref. ^51^ to demonstrate a fast non-volatile GST-based PCM memory with a capacity of 5 bits, occupying an area of only 4 × 1.3 µm^2^, while the speed was pushed close to 1 GHz in the all-optical memory cell presented in ref. ^52^ using only 13.4 pJ. As such, PCM-based optical memories offer some highly attractive benefits, which include (a) their small footprint^51^, (b) the broadband optical transparency^53^, (c) their ability to carry out the multi-bit and multi-level memory operation^52–54^, (d) their compatibility with silicon processing^55^, (e) the ultra-low energy requirements and, obviously, (f) their non-volatile nature, which has triggered a series of new and highly interesting applications, including their use as synaptic elements for neuromorphic computing architectures^94,95^. Their rather limited operational speed can be considered as among their drawbacks, although its non-volatile characteristics direct its employment in application fields where multi-GHz operation is not necessarily considered as a pre-requisite.

Of all the above proposed solutions, the VCSEL-based and SOA-based memory schemes present the most mature volatile optical memory implementations, as both have been well-established commercial optical technologies for more than two decades. With this taken into account and with consideration of the fact that the ultimate target for non-volatile memories is to reach the functional level of SRAM and DRAM circuitry, it is no surprise that the first demonstration of optical SRAMs^22^ was indeed accomplished with the mature SOA-based technology. Silicon- and PhC-based memory technologies have shown great potential to significantly improve the footprint, speed and energy consumption over VCSEL- and SOA-based memories and form the dominant volatile optical memory scheme of the future; however, they are both more recent technological platforms necessitating a few more steps to reaching the maturity level required by multi-bit optical RAM prototypes. When non-volatile optical memory setups are considered, this seems to be the stronghold of PCM-based optical structures, as they feature ultra-low footprint and multi-level memory capabilities that have already been successfully employed in several novel application fields^94–98^.

### From elementary optical memory cells to optical random access memories (RAMs)

The main performance metrics related to memory functionality and characteristics are summarized in Box 1, whereas Box 2 provides a brief overview of the different application segments that have been addressed thus far by optical memory technologies. Although non-volatile optical memories seem capable of penetrating their respective application targets without requiring any additional circuitry, most of the volatile memory application areas necessitate the employment of more advanced memory schemes with random access functionality on top of the simple storage mechanism. The random access operation goes beyond the simple latching operation offered by elementary volatile optical memories and is typically provided by SRAM^22–25,70,99^ and DRAM^48,93^ circuits, suggesting that volatile optical memory layouts have to proceed along their migration from elementary AOFFs toward complete optical RAMs with controllable access at any random time to form reliable alternatives for practical use in real application cases. This section provides a brief analysis of how elementary volatile memories can scale into RAM cell layouts by simply enriching their design with optical access gating functions.

**Fig. 5:**
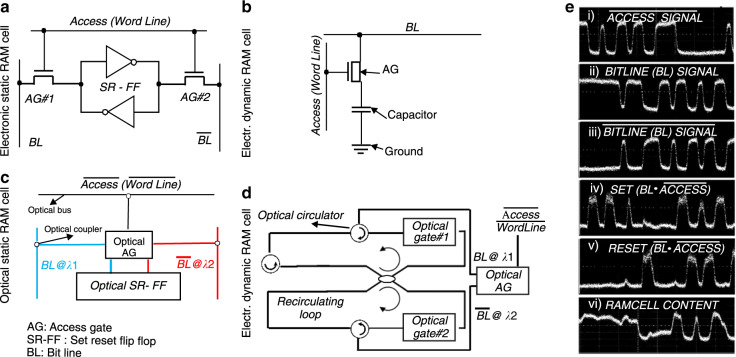
Electronic and Optical SRAM and DRAM architectures. **a** Electronic 6T SRAM cell, **b** optical SRAM cell implementation according to ref. ^22^, **c** electronic DRAM cell, **d** optical DRAM cell with recirculating fiber loops 48, and **e** pulse trace in write mode operation of optical SRAM cell at 10Gb/s: (top to bottom) (i) inverted access bit ($\overline {Access}$), (ii) incoming BitLine (BL) signal, (iii) inverted BitLine $\overline {(BL)}$, (iv) set and (v) reset signals, respectively produced as the AND product between BL and $\overline {BL}$with Access, and finally, (vi) RAM cell memory content

The first attempts toward deploying optical RAM cells were inspired by the architectural layouts of the respective electronic SRAM and DRAM cells. Figure 5a depicts the standard 6T electronic SRAM cell that comprises a bistable transistor-based structure typically made of two back-to-back connected CMOS inverters. These cross-coupled inverters form the AOFF mechanism, i.e., the SR-FF, and comprise the main memory cell used for bit storage. An AOFF, as the basic storage circuit, has two stable states (“logical zero” or “logical one”) that alternate through external input signals (i.e., set/reset in the case of SR-FF). However, to migrate to advanced RAM functionalities, additional gating circuitry is required apart from the AOFF to enable communication between a memory and its environment through the read/write functions. The random access operation is provided by two access transistors that are additionally employed as access gates (AGs), controlling the communication of the memory cell with the “outer world” depending on the applied access (word line) signal^100^, which designates whether the memory cell has been selected to perform the write or read functionality. Figure 5b depicts the standard electronic DRAM cell, which employs a simple capacitor instead of a latch as its main memory cell and one additional transistor that acts as an AG to activate the data. The capacitor is either charged or discharged, corresponding to the two possible data values (logical “1” or logical “0”).

Transferring these principles into the optical domain can result in the respective optical SRAM and DRAM cells, as shown in Fig. 5c, d, respectively. The optical SRAM cell utilizes an optical AOFF circuit (i.e., an SR-FF) as its main memory cell, which can be any of the volatile latching memories analyzed in the previous section, whereas the optical DRAM cell employs two coupled recirculating loops as its main memory element, as shown in ref. ^48^. In both RAM cells, the optical AGs are realized by ON–OFF optical switches controlled by the access (word line) bit, typically operating in inverted logic and necessitating their control by the inverted access signal in the thus far reported optical SRAM^22–25,70,99^ and DRAM^48,49,93^ implementations. This means that an access bit of logical “0” value blocks the transition of the optical BitLine (BL) and its inverted $\overline {BL}$ signal through these AGs, prohibiting communication between the memory cell and the outer world and allowing the optical RAM cell to retain its previous logical content. Access to the memory cell for the read/write operation is enabled only when the optical access bit becomes “1” and the respective $\overline {Access}$ signal becomes “0”. Read mode operation is accomplished when $\overline {Access}$ is set to “0” and no new bit values are carried by the BL and $\overline {BL}$ lines, allowing the memory content and its complementary value to reach the BL and $\overline {BL}$ buses, respectively. When the $\overline {Access}$ signal has a logical value of “0” and the write operation is targeted, a logical bit stream and its complementary signal are applied at the BL and $\overline {BL}$ lines, respectively. These signals enter the SRAM or DRAM cell through an AG and are inserted into the memory cell, acting as the corresponding set and reset signals. One of the main advantages of optical SRAM implementation over its electronic counterparts is the potential to employ different wavelengths for the optical BL and $\overline {BL}$ signals, which can allow the use of a single multi-wavelength AG element instead of the two AGs typically required in electronic SRAMs, as illustrated in Fig. 5c.

An example of the write operation at 10 Gb/s can be observed in Fig. 5e, which depicts the respective results of the recently successfully demonstrated optical static RAM cell^70^, which utilizes a monolithically integrated SOA-based SR-FF and an SOA-MZI-based dual-wavelength AG element. Figure 5e-i presents the pulse sequence of the optical $\overline {Access}$ signal, whereas Fig. 5e-ii, iii illustrate the optical BL and $\overline {BL}$ signal sequences, respectively. Figure 5e-iv, v reveal the set and reset signals, respectively, obtained after the AND operation between the BL and $\overline {BL}$ with the access bit and entering the memory cell. In the absence of an $\overline {Access}$ bit (logical state equal to “0”), successful write functionality can be confirmed by the SRAM cell content shown in Fig. 5e-vi, the logical value of which exactly follows the content of the incoming BL sequence. When the logical value of the $\overline {Access}$ becomes high (i.e., logical state equal to “1”), both the set and reset signals become zero, and the memory content preserves its previous state, remaining unchanged.

Although the transition from elementary memory units to complete optical SRAM and DRAM cells relies on the rather simple addition of optical AG elements, so far, only a few volatile AOFF structures, including coupled SOA-based modules^22–25,70^ and bistable semiconductor ring lasers^99^, have managed to scale to fully functional optical SRAM^22,23,70,99^ and DRAM^48,49^ layouts. Optical DRAM cell demonstrations^48,49^ have still not managed to escape the fiber-based landscape, but optical SRAM cell implementations^22,23,70,99^ are constantly adopting higher integration-density AOFF technology moving from hybrid-integrated^22^ to monolithically integrated^25,70^ configurations, reducing their footprint by 99.8% within the last ten years. They have also recently managed to break the electronic SRAM speed^101^, releasing the fastest SRAM cell reported thus far at 10 Gb/s^70^, whereas the demonstration of WDM-enabled SRAM circuitry^25^, together with the complete optical cache memory design^102,103^ and the experimental verification of the required optical cache memory peripheral circuits^104,105^, suggests a strong potential for fabricating complete optical cache memory prototypes in the near future and enabling completely new computing architectures, such as disintegrated computing with macrochips^106^. Moreover, the recent employment of monolithic InP-based AOFF technology for novel optical content addressable memories (CAMs)^107–109^ that were demonstrated to reach operational speeds almost 10× higher than those of the respective electronic CAMs implies that optical SRAMs could also form a viable vehicle with which to penetrate the routing look-up table application area when combined with the respective optical CAM technology^107–109^.

Box 1 Basic performance parameters of memory units The long-term experience obtained from using electronic memory devices outlines the following parameters as the most critical performance indicators for the practical perspectives of any optical memory technology:

**Memory bandwidth**

This parameter is defined as the rate at which data words can be retrieved from or stored in an optical memory. Today, state-of-the-art chip multiprocessors (CMPs) exhibit limited bandwidths of 20 GB/s^122^ and 8.5 GB/s that correspond to 160 Gb/s and 64 Gb/s for cache and DRAM access, respectively. At the same time, high-performance state-of-the-art SRAM line rates do not exceed 4–4.6 GHz^101,123^. Photonic solutions seem to hold the potential for higher bandwidth values but have thus far been restricted solely to interconnect implementations^124–126^.

**Energy efficiency**

This parameter is usually expressed in mW/Gb/s or pJ/bit at a certain operating frequency and emerges as a key factor toward developing multi-bit optical memories. Unlike electronic memories that require more energy to reach higher bit rates, optics offer bit-rate transparency, as their operating frequency is irrespective of the required energy.

**Integration density**

This parameter appears to be the most important factor when moving to high-capacity multi-bit memory banks and is also associated with the footprint of the device. Footprint is defined as the area occupied by a device, calculated as width × height in the case of a 1-bit memory cell. In electronics, the scaling of CMOS transistors has led to modern IC chips with more than one billion transistors, allowing for high-density SRAMs. For example, in state-of-the-art 22 nm technology^101^, a bit cell footprint of 0.092 μm^2^ can enable high array densities up to 6.7 Mb/mm^2^. High-density electronic SRAM bit cells have already been reported, achieving 0.027 μm^2^ and 0.031 μm^2^ bit cell sizes in 7 nm^119^ and 10 nm^127^ processes, respectively, corresponding to 23.6 Mb/mm^2^. The integration density in the case of optical memories is tightly related to the fabrication technology employed, but obviously, the miniaturization of photonics has not yet reached the maturity level of electronics, whereas the simultaneous optimization of the performance and footprint in optical memory layouts has just begun. Thus far, the highest integration density in optical memory cells reported is 128 b/mm^39^. Recently, PCM memories with a capacity up to 5 bits^51^ have been presented, occupying 4 × 1.3 μm^2^, corresponding to a potential integration density of 8 kb/mm^2^, whereas a plasmonic photonic memory^112^ has presented an unprecedented low footprint of 0.0025 μm^2^, holding the potential for even higher integration densities in the future.

**Access time**

This term is defined as the time delay between a request to access a data bit and the successful completion of that attempt. On an electronic memory cell basis, this time is dominated by the RC delay of the transistor employed in the memory architecture and can also be expressed as 1/(bit rate), representing the actual time required to switch-on the transistor. State-of-the-art high-performance 6T SRAM cells based on Tri-Gate technology exhibit an access time of ~200 ps. Moving to more complex systems (i.e. array level, system level), the access time increases to several ns as the access time accumulates delays due to different resistances and capacitances present in the memory system. Optical bit-level memory implementations already demonstrate access times <50 ps^30^, with the access time here denoting the response time of the optical memory cell unit without including, of course, any propagation time or any latency associated with additional functions.

Box 2 Applications of optical memory technologies Drawing from the vast experience with the diverse applications of electronic memory technologies, optical memories have gradually penetrated into multiple application sectors that include processing, routing, and computing, as follows:

**Optical digital signal processing and optical Boolean logic**

This area involves the deployment of the necessary optical building blocks for realizing the complete toolkit of elementary digital processing electronic circuitry directly in the optical domain, including among others of all different types of flip-flops, shift registers and counters. Set-reset flip-flops (SR-FFs) have been implemented by a great variety of bit-level optical memory technologies^22–36^, while more advanced D-type^32^ and toggling AOFFs^26,27,31,32^ have also been realized. Shift register and bit counter configurations that require the cascaded employment of multiple AOFFs have been demonstrated, mainly relying on VCSEL-^40,63^ and SOA-based^32,57^ memory setups. This application area primarily aims to replicate well-known functions and layouts at much higher operational speeds toward enabling true processing via optics. In addition to the performance parameters described in Box 1, this application segment requires a solid cascadability potential of the AOFF technology.

**Contention resolution and buffering in optical packet switches (OPSs)**

Contention resolution refers to the procedure carried out in packet switched networks to avoid a collision between incoming data packets that require, at the same time, the same router output port, which would result in non-recoverable signal degradation and subsequently in the loss of information. The most typical way to resolve contention relies on the use of buffers to delay one of the two contending packets until the desired outgoing port again has an available timeslot. Realizing optical buffers for storing optical packets in OPS fabric demonstrations was widely researched in the early 2000 s, often residing in the areas of recirculating fiber loops^3–5^ or fiber delay lines^6–9^ to circumvent the absence of true optical RAM buffers. However, these setups can offer only a limited buffering time; hence, the quest toward real optical RAM buffers soon became a necessity and was pioneered in a big R&D initiative in Japan^36–39,128^, promoting for the first time the use of InP photonic crystal nanocavities for packet buffering purposes in OPS fabrics. Alternative contention resolution methods have used optical FFs to provide alternative wavelengths toward triggering wavelength conversion circuits and resolution in the wavelength domain^12,13^.

**Address look-up table and forwarding**

These functions usually take place at a router to identify the destination address of the incoming data and to force the data to leave through the correct router outgoing port. Address look-up involves a procedure where the incoming address is compared with a set of possible addresses stored locally in the router, while forwarding indicates the procedure where the address matching is associated with the router outgoing port that has to be activated. Address look-up is typically performed by means of content addressable memories (CAMs) that comprise a special type of memory performing simultaneously memory and comparison operations within a single clock cycle^129^. Optical look-up tables have not yet been realized, but the recent demonstrations of optical binary^130^ and ternary^107^ CAM cells using coupled SOA-MZI-based FFs might release new perspectives for all-optical look-up table deployments. Forwarding is usually implemented by a 2-dimensional RAM bank, where every RAM row stores the address of a router output port and is activated by the look-up CAM-based table.

**Cache memories**

Caching is typically used in computing systems to store a small but often accessible amount of data close to the CPU to allow for ultra-fast fetching, avoiding the increased latency times of remote DRAM accesses. They exploit static RAM cells surrounded by certain peripheral circuitry for read/write control, row/column decoding and tag comparison purposes. The first all-optical cache design was proposed recently in refs. ^102,103^, while all its constituent building blocks were demonstrated experimentally^22–25,104,105^.

### Opportunities and challenges

**Fig. 6:**
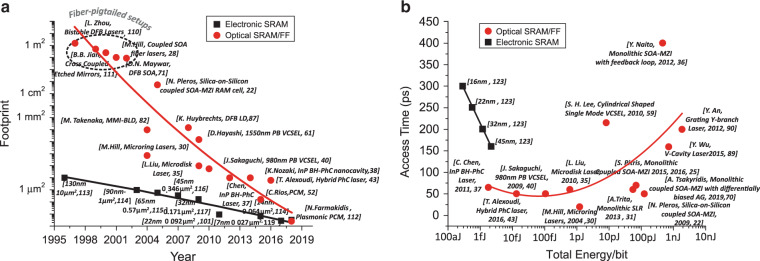
Evolution and comparison of optical and electrical memories. **a** Evolution of optical and electrical memory components in terms of footprint over the last two decades. **b** Memory access times in picoseconds versus total energy consumption per bit for both optical and electronic technology

The significant progress witnessed in elementary optical FF memory and optical RAM layouts in comparison with the respective evolutions in electronic SRAMs can be clearly overviewed in Fig. 6. Figure 6a depicts the size scaling over the last 25 years, indicating that the emergence of reliable photonic integration around early 2000 triggered some rapid footprint improvements in optical memory setups. Optical memory cells rapidly evolved from m^2^-scale layouts constructed with fiber-pigtailed bulk components^28,71,110,111^ until the early 2000s, reaching down to mm^2^-scale hybrid-integrated configurations when employing planar lightwave circuit (PLC) technology^22,68^ and then to the more compact μm^2^-scale monolithically integrated memory cells^25,70^ recently developed, promising sizes of sub-μm^2^ offered by volatile photonic crystal nanocavities^38,39^, nanolasers^37,43^ and the non-volatile PCM-based memory cells^52,112^. This implies a 12-orders-of-magnitude improvement in footprint scaling for optical memory devices during the last 20 years. Over the same period, electronic technology^113–119^ gradually moved from the 130 nm^2^ processing node^113^ down to the 7 nm^2^ node size over the same time period, reducing the SRAM cell size by 3 orders of magnitude and featuring an SRAM cell size of 0.027 µm^2^ ^119^. Despite this comparison being carried out while considering both smaller-size and simpler elementary memory elements together with complete SRAM cells for the optics with only SRAM cells being taken into account for electronics where the size is slightly higher compared to the incorporated electronic latch, it is still revealed that all-optical memory technology has made radical progress that continues to exhibit a steep improvement slope.

Figure 6b compares the relevant progress experienced by optical memories with respect to the memory access time and energy efficiency, again being compared with several generations of electronic SRAMs that typically comprise the fastest electronic memory layout. The optical memory setups again include the elementary volatile optical latching elements as well as more advanced optical SRAM cells reported thus far, without encompassing the non-volatile optical memories that are well known to support lower operational data rates compared to their volatile counterparts. In this graph, the power efficiency is expressed as energy/bit, taking into account both the required optical/electrical bias energy and the optical switching energy for the optical components, as reported in Table 1. For the electronics, the power efficiency has been deduced as the cell-level write energy including the energy of charging the storage node plus the energy of opening the two access transistors^100^. This graph clearly reveals that the power efficiency of optical memories has been drastically reduced by 5 orders of magnitude from the value of ref. ^99^ pJ/bit down to 1 fJ/bit as optical memory technology has moved from mm-long SOA to μm-long InP micro-rings, microdisks, and VCSELS and then further to III–V photonic crystal cavities. It is important to note that this energy efficiency improvement has occurred with a simultaneous reduction in the memory access time, highlighting the fact that optics can take advantage of their bit-rate transparent power consumption to offer high bit-rate capabilities simultaneously with improvements in the energy efficiency. On the other hand, electronic setups can hardly cope with simultaneous advances in both memory access times and energy efficiency. The speed-up of electronic gates comes at the cost of increased energy and heat dissipation requirements, finally restricting clock speeds to a few GHz to remain within a reasonable energy envelope^120^. Today’s electronic SRAM cells are severely challenged by the increasing standby leakage induced by the increased gate, sub-threshold, and junction leakage of the minimum-sized FETs^100,121^. To cope with the leakage-induced energy burden while keeping in line with Moore’s law, memory designers have resorted to sub-threshold voltage techniques in SRAM circuits that are accompanied by severe reliability issues during read/write operations; thus, the energy reduction comes at the cost of lower speed operation^121^. This is clearly captured in Fig. 6b, as the electronic power efficiency evolution line moves from the fJ regime of the 45 nm technology with access times of ~160 ps to the aJ regime enabled by the 16 nm technology, where, however, the access times increase to 300 ps^100^.This practice reveals that the race for lower-energy electronic SRAMs comes at the loss of access time improvements, whereas optics have proven their bit-rate-transparent power consumption even in the memory domain, reaching 50 ps access times and close to 1 fJ/bit energy efficiencies^37^, canceling in this way the trade-off between access time and energy efficiency. The fitting among the optical memory technology bullets in Fig. 6b forms an evolution line that points toward the desired target specifications for both low energy and low access times, whereas the respective electronic line seems to deviate from the low access time requirement to reach the low-energy target.

**Table 1:** Summary of optical memory technologies

Switching time (ps)		Freq. (GHz)	Energy efficiency (pJ/bit)				Footprint (μm^2^)/cell	Capacity (bits)	Technology/refs.
			Switching^a^	Static^b^		Total			
On	Off			Electr.	Opt				
20	20	50^c^	0.0055	2.16^d^	–	2.16	720^30^	1	Micro-ring lasers^30^
<50	<50	5^e^	~0.6	120	–	120.6	540 × 106^68^	1	Silica on silicon coupled SOA-MZIs^22,68^
50^f^	50^f^	40^e^	0.00475	0.1^f^	–	~0.105	36	4	1550-/980 nm PB VCSEL^40,41^
60	~100	10^g^	0.0018	0.6^f^	–	0.6	56.25^35^	1	Microdisk laser^35^
58	65	~15^c^	0.00031	–	0.0017	~0.002^h^	<10	1	BH-InP PhC nanolaser^37^
44	7 × 103	0.142^i^	0.0025	–	0.00021	~0.0027	<10	104/128	BH-PhC nanocavity^38,39^
200	200	5^c^	~0.6	~450	–	~450.6	40 × 10^6^	1	Monolithic SOA-MZI with feedback loop^36^
10	60	10^e^	~18	36	–	~54	0.03 × 10^6^	1	Semiconductor ring laser^33^
70	70	10^e^	3	~120		~123	12 × 10^6^	1	Monolithic coupled SOA-MZIs^25^
25	75	10	0.5	~180		~180.3	12 × 10^6^	1	Monolithic coupled SOA-MZIs with differentially biased push-pull technique^70^
50	50	10^e^	0.0032	–	0.01	0.013^h^	6.2	1	III–V on SOI PhC nanocavity laser^43^
10 × 103	10 × 103	1^g^	5.3	–	–	5.3	0.16–0.25	3	PCM^52^

^a^Based on the reported switching energy or calculated as the product of the switching power and the pulse duration

^b^Calculated as the product of the electrical/optical power and frequency (pulse duration). Electrical static power is noted for electrically pumped memories, whereas optical static power refers to the bias power required in the case of optically pumped memories

^c^Frequency estimated by the authors of the current work based on the reported response time

^d^Based on the calculation provided in ref. ^35^, we estimated the electrical consumption for both micro-ring lasers (2 × 30 mA assuming 1.8 V with 20 ps pulses) excluding wavelength tuning

^e^Frequency demonstrated by the authors in their respective papers

^f^Based on the performance table of ref. ^38^. The total energy excludes the wavelength tuning energy consumption

^g^Frequency reported by the authors in their respective papers

^h^Excluding wavelength tuning energy consumption

^i^Frequency estimated based on the switch-OFF time, which equals 7 ns according to refs. ^38,39^

^9^Based on the performance table of ref. ^38^ and including the wavelength tuning energy consumption. The overall power consumption according to ref. ^53^ is estimated to be 6 mW (3.5 mA × 1.5 V + 0.8 mW for wavelength tuning)

These evolution trends highlight the future perspectives of optical memory technology to meet the complete framework of performance requirements along a broad field of volatile memory applications: high integration densities and, as such, high-capacity modules, low memory access times and low-energy consumption. Turning this promise into a tangible reality has, however, still to overcome some pressing challenges to confirm the use of this light-enabled memory roadmap in real computing, signal processing, routing, datacom and telecom applications:

- Theoretical predictions about the volatile memory line-rate operation up to or even beyond 40 Gb/s^56^ have to be experimentally confirmed through a low-footprint, low-energy and fast-access-time photonic integrated circuit technology, concluding with speed-optimized integrated memory layouts.
- The potential for high-integration density and small-footprint setups has to be validated in high-memory-capacity configurations, elevating current optical memory capacity metrics from just a few bytes to kB or even Mb implementations. This obviously requires an intense effort to improve the yield of photonic integrated memory circuits while optimizing the architectural layout to reduce possible undesired thermal stability and crosstalk effects.
- Low-footprint, low-energy and high-speed-credible integrated optical memories have to take the next step in evolving from simple memory elements into highly functional and practical optical SRAM, DRAM and CAM cells, which have been reported thus far only via the use of SOA-based technologies.
- Low-footprint and energy-efficient non-volatile PCM-based optical memories need to (a) overcome materials science issues related to the stability of the amorphous phase that defines the data retention in memory devices and (b) improve the crystallization time to achieve faster operation in excess of a few GHz.
- Finally, their integration roadmap has to be shaped around a high-yield and low-cost fabrication technology allowing for dense optical memory architectures to arrive at scales, complexities and cost-efficiencies similar to those of their electronic counterparts.
